# Antipsychotic deprescribing as a problem of care, not compliance

**DOI:** 10.1038/s41537-026-00745-y

**Published:** 2026-04-21

**Authors:** Helene Speyer, Marte Ustrup, Mette Ødegaard

**Affiliations:** 1 CORE research unit. Mental Health Center Copenhagen, Copenhagen, Denmark; 2 Faculty of Health and Medical Sciences, University of Copenhagen, Copenhagen, Denmark; 3 Unit for Complicated Schizophrenia, Mental Health Center Glostrup, Glostrup, Denmark

**Keywords:** Diseases, Schizophrenia

We read the new article collection in Schizophrenia on antipsychotic discontinuation with interest, but also with concern regarding both its framing and scope. In the main review article, Moncrieff and Horowitz outlines the rationale and evidence^1^, followed by five commentaries^2–6^. Our perspective here comes from what can be described as our learned, labored, and lived experience with antipsychotic discontinuation^7,8^.

Our first concern relates to the breadth of perspectives represented in this collection, which appears limited. The majority of the contributors largely reflect a similar demographic and professional profile, with career trajectories shaped within the same institutional and commercial contexts. Notably absent are people with lived experience of long-term antipsychotic use and discontinuation, as well as broader forms of experiential diversity. This absence is not merely representational; it has epistemic implications^9^. Lived experience contributes forms of knowledge directly relevant to withdrawal phenomena, relapse, side effects, identity, and recovery. When such perspectives are largely excluded, there is a risk that professional risk management comes to dominate over experiential understandings of harm, benefit, and valued outcomes. In Leucht and Rodorico’s paper, the authors try to imagine what it is like: *“If we put ourselves in a patient’s shoes, we might cautiously and very slowly… want to explore whether we are among the approximately 20% who do not relapse…, taking into account the circumstances of our first episode.”* Future work might benefit from directly involving people with lived experience in such reflections. They continue: “*Additionally, we aim to collect the experiences, tips and tricks of physicians, to effectively “co-create the guideline with its users*.”^5^ We do hope, that what is meant here, is to co-create with those who this is about in the future, rather than limiting the co-creation process other physicians.

Second, the commentaries raise important questions about how medical recommendations are conceptualized. While the primary review^1^ provides a structured synthesis of the literature, several responses move toward prescriptive statements about what clinicians should recommend, implicitly positioning clinicians as decision-makers regarding discontinuation. We believe this is evident in Zipursky et al.’s comment: “*Given the extremely high risk of psychotic relapse in schizophrenia and the severe sequalae that often result, the mental health and physical health benefits of long-term antipsychotic treatment should be expected to outweigh the risks for the large majority of people with schizophrenia*”^2^. And further in Correll et al.’s comment: *“Antipsychotic discontinuation after established acute benefits should be the exception rather than the rule in people diagnosed with severe forms of psychotic disorders, such as schizophrenia”*^6^.

In ethical clinical practice however, clinicians do not decide on behalf of patients; they provide information, care, and relational support to enable informed choices. In contexts characterized by substantial uncertainty and heterogeneity, binary recommendations for or against discontinuation have limited conceptual validity. Individuals differ markedly in how they value relapse risk, medication side effects, autonomy, and quality of life^10,11^. Evidence alone does not determine decisions; it informs shared deliberation^11^ that integrates empirical findings with individual preferences, histories, and goals. As such, “evidence-based” decision regarding antipsychotic discontinuation must be expected to vary between people, rather than to be reduced in one-size fits-all recommendations. Embracing this heterogeneity and supporting people in making the *for them* right decisions are where the key to recovery-oriented care lies^12^.

The main argument for continuous maintenance treatment rests on reduced relapse rates. However, relapse has been defined in multiple ways across trials^13^. Moreover, relapse frequency does not necessarily capture long-term functional outcome or subjective recovery, as illustrated by long-term follow-up studies in which short-term relapse differences did not translate into poorer long-term functioning^14^. The observation that most people discontinue antipsychotic medication^15^, often repeatedly over the course of treatment, should be treated as a meaningful global outcome^16^ reflecting the balance of effectiveness, tolerability, and acceptability in real-world settings^17^, rather than as a simple marker of non-compliance. From a patient perspective, discontinuation is rarely a trivial or uninformed act; rather, it typically reflects an accumulation of reasons that outweigh perceived benefits, including side-effect burden, impact on identity and functioning, and a desire for greater autonomy^18,19^. When viewed in this light, patterns of repeated discontinuation can be understood as signals of how treatment is experienced over time, integrating dimensions that are not fully captured by symptom scales or clinician-rated adverse event measures. From this standpoint, discontinuation warrants careful clinical attention not only as a risk factor for relapse, but also as a source of insight into the lived tolerability of long-term antipsychotic treatment. Thus, the relevant question is not whether discontinuation should be recommended or not, but how people can be supported as safely as possible when they wish to explore whether they still require medication^10^. This includes careful and flexible tapering practices, psychosocial support, monitoring for early warning signs, advance planning for crises, and accessible pathways for treatment re-escalation if needed. Such an approach neither denies relapse risk nor assumes that discontinuation is benign but supports the dignity of risk taking^20^.

In Denmark, patients have a legally protected right to receive clinical support during medication tapering, even when clinicians disagree with the decision to reduce or discontinue treatment^21^. Specialized tapering clinics have been established for people diagnosed with schizophrenia. Early clinical experience suggests that carefully supported tapering can be undertaken without severe adverse consequences and without evidence of inducing treatment resistance^22,23^. The first study from the Danish clinic reported that of the 88 patients included, 22 (27%) experienced relapse during the six-month tapering period, while 29 (37%) experienced relapse at the 12-month follow-up visit and nine patients were antipsychotic free. Following relapse, patients were clinically stabilized and showed an improved attitude toward antipsychotic medication^17,22^. While such observations do not replace systematic research, they may indicate that alternative clinical approaches are feasible and ethically coherent. On a practical level, psychiatrists can refer individuals to the clinic based on their wish to reduce medication, even if psychotic symptoms are present, provided there is no acute risk. At the clinic, a different psychiatrist conducts an assessment and, if appropriate, offers a gradual and individualized tapering plan, that can be adjusted accordingly. During tapering individuals have weekly appointments with a nurse, and monthly appointments with a doctor. Individuals may remain affiliated with the clinic for up to 12 months after successful tapering/discontinuation, depending on clinical needs and preferences.

Taken together, these considerations suggest that the central issue is not whether a risk of relapse exists, but how healthcare systems respond to uncertainty. A right-based, recovery-oriented approach acknowledges that individuals may choose to explore medication reduction despite potential risks. In this context, the role of clinicians is to support informed decision making, enhance safety, and remain engaged throughout the process, rather than dismiss or refuse patients’ wishes, which some studies suggest can occur in clinical practice^24^.
